# Risk Perception of Traffic Accidents Due to Alcohol and Marijuana Use in Mexican College Students

**DOI:** 10.3390/healthcare11071009

**Published:** 2023-04-01

**Authors:** Alberto Jiménez, Bruna Brands, Robert Mann, Gabriela Saldivar, Angélica Juárez-Loya, Pamela Garbus, Catalina González-Forteza

**Affiliations:** 1 Epidemiological and Psychosocial Research Department of Instituto Nacional de Psiquiatría Ramón de la Fuente Muñiz, Tlalpan 14370, Mexico; alberj@imp.edu.mx (A.J.); saldivh@imp.edu.mx (G.S.); catiartes@gmail.com (C.G.-F.); 2 Department of Pharmacology and Toxicology, University of Toronto, Toronto, ON M5S 1A8, Canada; bruna.brands@camh.ca (B.B.); robert.mann@camh.ca (R.M.); 3 Clinical and Health Psychology Department, Psychology Faculty, Universidad Nacional Autónoma de México (UNAM), Mexico City 04510, Mexico; 4 Cerro de las Campanas, Centro Universitario, Psychology Faculty, Universidad Autónoma de Querétaro, Santiago de Querétaro 76010, Mexico; pamela.garbus@uaq.mx

**Keywords:** driving under the influence of drugs, marijuana, alcohol, risk perception, young people, college students

## Abstract

Driving under the influence (DUI) of alcohol and other drugs is a common occurrence in Western societies. Alcohol consumption is related to 15% of fatal injuries in traffic accidents worldwide, with those DUI of alcohol being up to 18 times more likely to be involved in a fatal accident. Evidence for DUI of alcohol or marijuana among the college population in Mexico is scarce. This research estimates the proportion of use of alcohol and marijuana, describes the risk perception of DUI, and evaluates the relationship between risk perception and DUI behaviors in a sample of Mexican college students aged 18 to 29. The study was cross-sectional with a non-probabilistic sample. Risk perception of suffering traffic accidents when DUI or riding with someone DUI of alcohol, marijuana, or both, was high, unlike the risk perception of being detected or sanctioned for a DUI of marijuana. The study provided valuable information on the risk perception of engaging in behaviors related to DUI of alcohol and/or marijuana. It is necessary to undertake research on the subject with probabilistic and representative samples of this population of Mexico.

## 1. Introduction

Driving under the influence (DUI) of alcohol and other drugs is a common occurrence in Western societies [1]. Alcohol consumption is related to 15% of fatal injuries in traffic accidents worldwide [2], with those DUI of alcohol being up to 18 times more likely to be involved in a fatal accident [3]. Marijuana is the most frequently used illegal drug worldwide [4] and the one most used by drivers who drive intoxicated [5]; those who DUI of marijuana are at twice the risk of accidents [6].

Injuries caused by traffic accidents are the leading cause of death worldwide and the second main cause in Latin America in the group aged five to 29 [2]. Alcohol and marijuana use rates are high among young people in this region [7], putting this population at risk of suffering traffic accidents under the influence of these drugs [8].

Traffic accident deaths have a high rate in countries with emerging economies [2]. One of the most dangerous behaviors linked to drug use is DUI. Its prevalence among the youth population has been reported in several countries, and it is known that driving under the influence of alcohol and marijuana is one of the main causes of injuries and deaths due to traffic accidents [9].

Some studies have reported that the proportion of college students who have driven after consuming alcohol is between 15% and 43%, while the percentage of those who have done so after using marijuana is between 13% and 53% [10,11]. In other cases, rates of DUI of marijuana are the same or higher than those for DUI of alcohol [12].

Risk perception of suffering traffic accidents from using marijuana and DUI is low in young people [13,14] and people with low-risk perception and those who use marijuana are more likely to DUI [14]. Young people who drive after using marijuana believe they are less likely to experience negative consequences, such as being detected by the police, than those who DUI of alcohol [13]. This may be because the instruments for detecting marijuana use by drivers are still only rarely used [15,16].

Combined alcohol and marijuana use synergistically increases the risk of harm. Drivers who use these drugs at low levels may believe they are using them in moderation, but deterioration is more acute when substances are used together [9]. Although combined use appears to be quite common, more research is needed.

Evidence for DUI of alcohol or marijuana among the university population in Mexico is scarce, while the combined effect of these drugs on driving a motor vehicle has barely been explored in other countries [10]. Some studies have reported that 8% of Mexican students have problematic alcohol use, with 17% engaging in alcohol abuse [17], with a prevalence of risky and harmful consumption of 11% and 18% respectively [18]. According to the Monitoring the Future Survey, 44% of college students reported using marijuana in the past year, and 8% reported using marijuana every day [19].

There is an association between DUI of alcohol and/or marijuana and traffic accidents. Evidence shows that the risk perception of using drugs and the attendant negative consequences may contribute to increase consumption and escalate related problems. Although there is evidence of risk factors and perceptions of DUI of drugs; more information is required for public policy and prevention, particularly regarding DUI of alcohol, marijuana, and the two combined [13]. Studies in Mexico on DUI of alcohol and/or marijuana are scarce. This study seeks to generate evidence of these problems. The objectives of this work are: to estimate the proportion of use and problematic use of alcohol and/or marijuana, describe the risk perception of DUI and riding in a vehicle driven by someone DUI of alcohol and/or marijuana, and evaluate the relationship between risk perception and DUI behaviors in a sample of Mexican college students aged 18 to 29.

## 2. Materials and Methods

### 2.1. Research Design

The study was cross-sectional. A trained team of psychologists collected data using a self-administered questionnaire for college students in their classrooms. Data were collected in 2016–2017.

### 2.2. Population and Sample

The sample was non-probabilistic and selected using a three-stage procedure: (1) a random selection of five out of fifteen faculties was conducted; (2) at each faculty, groups were randomly chosen and (3) students found in each of the chosen groups were invited to participate; there were no rejections and all of the students who accepted concluded the questionnaire (100% response rate). The sample size was obtained with SurveyMonkey software. The calculation was based on the total number of students enrolled in face-to-face programs at the university; we used this parameter because the number of students within the age range was unknown. The sample included 507 undergraduate students from the largest university in Mexico.

### 2.3. Inclusion Criteria

Being enrolled in a face-to-face undergraduate or graduate program and being aged between 18 and 29.

### 2.4. Measurement and Instruments

The questionnaire included six sections and 59 items. To evaluate the dependent variable, behaviors related to DUI of alcohol and/or marijuana, items were adapted from the Ontario Student Drug Use and Health Survey (OSDUHS) [20]. They inquire about the frequency of use in the past year and whether students have driven or ridden in motor vehicles driven by someone under the influence of alcohol and/or marijuana. Possible answers include “Never”, “Once”, “Twice”, “Three times” and up to “Eight or more times”. These options were dichotomized, for analysis purposes, as “Yes” when participants’ answers ranged from “Once” to “Eight or more times” and “No” when the answer was “Never”.

To measure the independent variable, risk perception associated with DUI of alcohol and/or marijuana, a series of questions divided into categories were developed: risk of detection, risk of sanctions, and risk of an accident. These statements refer to possible events associated with DUI of alcohol or marijuana. For example: “How likely do you think it is that a policeman in your city will detect a driver driving under the influence of alcohol and/or marijuana?” Each item requires participants to rate their level of perception of the likelihood of occurrence, using a four-option Likert scale: “Very unlikely”, “Unlikely”, “Likely”, and “Very likely”; these options were dichotomized as “Unlikely” for those who answered the first two options and “Likely” for those who answered the last two options. Due to the nature of the questions, a higher likelihood indicates a higher level of risk perception.

To estimate alcohol and marijuana use, items were adapted from the CICAD/OAS (2019) survey that has been used in studies in Latin America and the Caribbean. These are dichotomous questions (yes/no) investigating alcohol and/or marijuana use during the past year and month.

The AUDIT (Alcohol Use Disorders Identification Test) was used to measure problematic alcohol use. It consists of 10 items that identify risky and harmful use patterns, as well as alcohol dependence, in the past 12 months. It is a valid, reliable scale, with high internal and test–retest consistency scores, and correlates with other measures used to assess problematic alcohol use; the scale has proven suitable psychometric properties in the Mexican population [21]. Answers to the first eight items are scored on a numerical scale from 0 to 4, and the last two items have only three response options (0, 2, 4). The scores of all the items are added to obtain a total score for interpretation [22]. In this study, a score of eight or more was considered an indicator of problematic use.

Problematic marijuana use was measured using CAST (Cannabis Abuse Screening Test), which has six items [23] assessing use in the past 12 months. Answers are scored using a numerical scale (0 to 4) that is added to obtain the total score (0–24). In this study, a score of three or over was considered an indicator of problematic use.

The questionnaire also included questions to gather demographic information such as age, sex, whether participants drive motor vehicles, and whether they possess a valid driving license.

### 2.5. Data Analysis

Data were analyzed using descriptive and inferential statistics. The former was used to determine the characteristics of the sample with respect to the variables of age, sex, prevalence, and type of use. The analyses included frequency distribution, central tendency, and dispersion (mean, median, and standard deviation) measures where appropriate. Chi-square analyses and contingency tables were used to evaluate the relationship between risk perception and behaviors related to DUI of alcohol and/or marijuana. Tables present differences in the sample sizes because students responded heterogeneously to each variable.

### 2.6. Ethical Considerations

The study was approved by the Center for Addiction and Mental Health and the Instituto Nacional de Psiquiatría Ramón de la Fuente Muñiz ethics committees. Students were given an informed consent form (IC), which they read and signed. The IC specified that: (a) their participation would be voluntary and that they could withdraw at any time, (b) their participation would be anonymous and no identifying information would be linked to their questionnaire, (c) they would not receive direct benefits for their participation, (d) their participation in the study implied low risk and (e) agreeing to participate or refusing to do so would not affect their grades. The students kept a copy of the IC, which included information on the researcher in case they had any questions or required additional information. To protect the anonymity of the participants, they were not asked to write their names on the questionnaires. This information was requested in the ICs, but the forms were stored in a different container from the questionnaires to ensure that the names could not be linked to the completed questionnaires.

## 3. Results

The sample had 507 participants (66% women, and 34% men). Alcohol use in the past year was reported by 83% and marijuana use by 16%. Of these, 17% were classified as problematic alcohol use and 19% as problematic marijuana use (Table 1).

Risk perception of being detected while DUI of alcohol was 56%, of being sanctioned was 52%, and of suffering a traffic accident was 99%. Risk perception of being detected while DUI of marijuana was 24%, of receiving a sanction was 36%, and of suffering an accident was 83%. Risk perception of being detected while DUI of both drugs was 73%, of being sanctioned was 71%, and of suffering an accident was 97% (Table 2).

Of those who reported the use of alcohol, marijuana, or both, 14% said they had DUI of alcohol while 48% had been a passenger in a vehicle driven by someone under the effects of alcohol. Five percent have DUI of marijuana while 11% have ridden in a vehicle driven by someone who used it before driving. Seven percent reported having DUI of alcohol and marijuana while 9% have ridden with someone who had driven after having used both (Table 3).

The comparison of risk perception behaviors related to DUI of alcohol, marijuana, or both, showed statistically significant differences in the likelihood of perception of receiving a sanction in the event of DUI of alcohol (*x*^2^ = 3.95, *p* < 0.05) and of probably suffering a traffic accident when riding as a passenger with someone who drives after having used marijuana (*x*^2^ = 3.67 *p* < 0.05) (Table 4).

## 4. Discussion

The prevalence of alcohol use among the general adult population was 56%, and dependence was 7% [24]. The proportion of alcohol use in the sample of this study during the previous year was 83%, and 17% of these scored for problematic use. Although the data are not entirely comparable, since this is a sample of college students and our estimate measured problematic use rather than dependence, it gives an idea of the magnitude of alcohol use and problematic use within this population. These data also coincide with others in which it has been reported that problematic use or abuse is between 8% and 17% and risky or harmful use is between 11% and 18% among university students [17,18].

The proportion of marijuana use in the past year in the sample was 16%, and 19% of these scored for problematic use. Data of use in the past year are higher than national rates for the adult population (2.1%) [24], but lower than those reported in other college populations (44% reported using marijuana in the past year, and 8% reported using marijuana every day) [19]. Trends of marijuana use in the past year have been rising steadily during the last twenty years in Mexico [24], which concurs with international trends [25].

Data on risk perception showed that it is very high regarding the likelihood of suffering an accident, with between 80% and 99% of the sample reporting that there is a risk when DUI of alcohol, marijuana, or both. Conversely, the risk perception of being detected was lower (56% for alcohol, 24% for marijuana, and 73% for both drugs). Other studies on young people and university students have reported the existence of a risk perception of suffering traffic accidents if DUI of alcohol [26] and, in smaller proportions, if DUI of marijuana [14]. There were also high proportions of risk perception of suffering an accident when riding with someone DUI. Between 72% and 99% of the sample said that it is possible to suffer a traffic accident in these circumstances. The proportions of risk perception of being detected when riding with someone DUI were lower (between 22% and 73%).

Risk perception was not related to the behaviors of DUI of alcohol, marijuana, or both combined. In the case of those who reported using alcohol, their risk perception of receiving a sanction was related to DUI of alcohol when they are the ones behind the wheel; this result makes sense considering the breathalyzer strategy implemented by the city government (Conduce sin Alcohol/Drive without Alcohol) since 2003 which aimed at reducing the incidence of DUI of alcohol. It is noteworthy that risk perception in those who consumed alcohol and have driven under the influence of alcohol, was significantly different in the perception of receiving a sanction since the evidence shows that DUI of alcohol is related to traffic accidents [3,9,14]. For the remainder of the sample, risk perception of suffering a traffic accident was related to riding as a passenger of someone DUI of marijuana.

The proportions of use and problematic use of alcohol and marijuana were higher in this sample than those reported nationally and in some studies on college students. Risk perception of suffering traffic accidents when DUI or riding with someone DUI of alcohol, marijuana, or both, was high, unlike the risk perception of being detected or sanctioned for DUI of marijuana.

Although the risk perception of suffering an accident was high, this was not related to DUI behaviors. In other words, although participants in this study are aware of the risk entailed by DUI, this does not necessarily mean that it has a negative impact on their likelihood of engaging in DUI of these drugs. These findings are important in that they reflect the emergence of a pattern of high-risk behavior for health and highlight the need to work with elements that involve the translation of risk awareness into the modification of behaviors that affect the well-being of this population group and may have a specific impact in both individual and public health terms, especially for young people.

## 5. Conclusions

This study made it possible to obtain valuable information on the risk perception of engaging in behaviors related to DUI of alcohol or marijuana. However, it is worth mentioning that it has certain limitations regarding its design. Given that we worked with a non-random sample, the results only offer an approximation and an initial description of a problem that is rising in Mexico and on which there is very little empirical information. It is necessary to undertake more research on the subject with designs that allow for more robust comparisons and measurements of the real effect DUI has on alcohol, marijuana, and the combination of both injuries and mortality due to traffic accidents in different population groups, with an emphasis on the youth population. These designs should include probabilistic and representative samples of this population of the country, given that a large sector of young people are not part of the student community and are exposed to DUI of these drugs or engaging in similar behaviors.

## Figures and Tables

**Table 1:** Sociodemographic data ^1^.

Variable	%	*n*
Age		
18–21	88.8	445
22–25	9.8	49
26–29	1.4	7
Sex		
Male	34.1	173
Female	65.9	334
Drive a motor vehicle		
Yes	32.7	166
No	67.3	341
Valid driver’s license		
Yes	34.1	173
No	65.9	334
Alcohol use in the past 12 months		
Yes	82.6	419
No	17.4	88
Marijuana use in the past 12 months		
Yes	16.2	82
No	83.8	425
Alcohol with marijuana in the past 12 months		
Yes	15.8	80
No	84.2	427
Problematic alcohol use	16.7*	68
Problematic marijuana use	18.8*	15

^1^ Data ONLY for those who reported alcohol and marijuana use.

**Table 2:** Risk perception of DUI.

Variable	Risk Perception			
	Likely		Unlikely	
	%	*n*	%	*n*
Alcohol				
Detection	56.1	284	43.9	222
Sanction	52.2	264	47.8	242
Damage	99.0	498	1.0	5
Marijuana				
Detection	24.3	123	75.7	383
Sanction	36.0	182	64.0	324
Damage	83.3	418	16.7	84
Both				
Detection	72.9	369	27.1	137
Sanction	70.9	358	29.1	147
Damage	97.0	491	3.0	15

**Table 3:** Participation in DUI behaviors.

Variable	Participates in DUI behaviors			
	Yes		No	
	%	*n*	%	*n*
DUI Alcohol				
Driver ^†^	14.0	55	86.0	339
Passenger	47.8	240	52.2	262
DUI Marijuana				
Driver ^†^	5.2	4	94.8	73
Passenger	10.7	50	89.3	416
DUI Both				
Driver ^†^	7.0	3	93.0	40
Passenger	8.6	40	91.4	425

^†^ Data ONLY for those who reported alcohol and marijuana use.

**Table 4:** Risk perception and DUI-related behaviors *.

Variable	DUI Alcohol %			DUI Marijuana %			DUI Both %		
	Yes	No	*x* ^2^	Yes	No	*x* ^2^	Yes	No	*x* ^2^
*Driver* ^†^									
Detection									
Likely	61.8	56.6		25.0	27.4		66.7	75.0	
Unlikely	38.2	43.4	0.33	75.0	72.6	0.00	33.3	25.0	0.00
Sanction									
Likely	69.1	53.7		25.0	37.0		66.7	65.0	
Unlikely	30.9	46.3	3.95 **	75.0	63.0	0.00	33.3	35	0.00
Accident									
Likely	100	98.5		50.0	80.6		66.7	100	
Unlikely	0.0	1.5	0.07	50.0	19.4	0.69	33.3	0.0	2.92
*Passenger*									
Detection									
Likely	58.3	53.4		22.0	25.5		72.5	73.4	
Unlikely	41.7	46.6	1.03	78.0	74.5	0.13	27.5	26.6	0.00
Sanction									
Likely	54.2	50.8		32.0	36.1		72.5	70.8	
Unlikely	45.8	49.2	0.45	68.0	63.9	0.17	27.5	29.2	0.00
Accident									
Likely	98.7	99.2		72.0	84.0		97.5	96.7	
Unlikely	1.3	0.8	0.01	28.0	16.0	3.67 **	2.5	3.3	0.00

^†^ Data ONLY for those who reported the use of alcohol, marijuana, or both. * Questions about risk perception were presented to all participants independently of their participation in DUI behaviors. ** *p* < 0.05.

## Data Availability

Data available on request due to privacy.
